# Interconnected idioblasts in *Peltaea polymorpha*: a novel component of the mucilage-secretory apparatus in Malvaceae

**DOI:** 10.1093/aobpla/plae063

**Published:** 2025-01-10

**Authors:** Tatiane Maria Rodrigues, Aline Rodrigues de Almeida, Juan de Nicolai, Igor Soares dos Santos, Silvia Rodrigues Machado

**Affiliations:** Department of Biodiversity and Biostatistics, Institute of Biosciences of Botucatu, São Paulo State University (UNESP), Professor Antônio Celso Wagner Zagnin street, 250, District of Rubião Júnior, 18618-970, Botucatu City, São Paulo State, Brazil; Interunit Postgraduate Program in Plant Biology, Institute of Biosciences of Botucatu and Rio Claro, São Paulo State University (UNESP), Brazil; Department of Biodiversity and Biostatistics, Institute of Biosciences of Botucatu, São Paulo State University (UNESP), Professor Antônio Celso Wagner Zagnin street, 250, District of Rubião Júnior, 18618-970, Botucatu City, São Paulo State, Brazil; Interunit Postgraduate Program in Plant Biology, Institute of Biosciences of Botucatu and Rio Claro, São Paulo State University (UNESP), Brazil; Interunit Postgraduate Program in Plant Biology, Institute of Biosciences of Botucatu and Rio Claro, São Paulo State University (UNESP), Brazil; Department of Biodiversity and Biostatistics, Institute of Biosciences of Botucatu, São Paulo State University (UNESP), Professor Antônio Celso Wagner Zagnin street, 250, District of Rubião Júnior, 18618-970, Botucatu City, São Paulo State, Brazil; Interunit Postgraduate Program in Plant Biology, Institute of Biosciences of Botucatu and Rio Claro, São Paulo State University (UNESP), Brazil

**Keywords:** Interconnected secretory cells, mucilage secretion, *Peltaea polymorpha* (A. St.-Hill) Krapov. & Cristóbal, rowed idioblasts, transversal walls

## Abstract

The anatomical and cytological characteristics of the mucilage-secretory system have been widely studied in Malvaceae. However, conflicting information regarding the morphological nature of secretory structures exists, and some remain poorly understood. In this sense, some secretory structures in Malvaceae are not characterized as typical isolated idioblasts, canals, or cavities. Here, we describe a novel component of the mucilage-secretory apparatus in the Malvaceae family. Samples of the shoot apex, mature stem and fully expanded leaves were obtained from adult *Peltaea polymorpha,* which grow in the Cerrado (Brazilian savanna). The samples were processed using standard light and transmission electron microscopy methods. Mucilage cells occurred in the cortex and pith of petioles and stems, and in the midrib of leaves. These cells originate early in the stem apex from successive divisions of cells of the fundamental meristem, resulting in a row of interconnected secretory cells enveloped by a sheath of parenchyma cells devoid of secretory activity. Mucilage is stored in both protoplast and apoplast. In the same row, some cells filled with mucilage become very swollen and compress the neighbouring idioblasts that become flattened. This phenomenon results in a sandwich panel structure consisting of the swollen transversal walls of adjacent cells. As the differentiation progresses, the transversal walls of the rowed mucilage cells became very swollen, multilayered, and porous. Cytoplasmic strands cross such transversal walls connecting rowed cells. Mucilage-secreting cells in *P. polymorpha* are interconnected idioblasts and represent a novel component of the mucilage-secretory apparatus in Malvaceae. These findings open new avenues for understanding the structure and dynamics of mucilage-secreting cells from a functional perspective.

## Introduction

Mucilage is a matrix of high molecular-weight compounds secreted in the form of a viscoelastic gel rich in polysaccharides (Sasse *et al*. 2018). Several functions have been attributed to mucilage, such as adhesion, lubrication, neutralization of chemical agents and pollutants, maintenance of the ionic balance of plant cells, source of nutritional resources, water retention, tolerance to freezing, wound healing and protection of the plant body against solar radiation and herbivory (Fahn 1979; Gregory and Baas 1989; Roshchina and Roshchina 1993; Vignesh and Nair 2018), which are common in xeromorphic species (Zaman and Padmesh 2009). Mucilage is an odourless, colourless, and tasteless substance with emerging commercial potential in the agricultural, food, cosmetic and pharmaceutical industries owing to its non-toxic and biodegradable properties (Galloway *et al*. 2020; Tosif *et al*. 2021; Cakmak *et al*. 2023; Goksen *et al.* 2023). Mucilage extracted from different plant species has been commercially exploited for the production of emulsifying, stabilizing and thickening agents owing to its functional properties (Tosif *et al*. 2021), and the quantity and composition of the mucilage produced determines its eventual usefulness (Vignesh and Nair 2018).

Mucilage solitary cells, secretory cavities and canals have been reported in the leaves, wood and bark of diverse plant families, including the Malvaceae (Solereder 1908; Metcalfe and Chalk 1950; Bakker and Gerritsen 1989; Gregory and Baas 1989; Clifford *et al*. 2002; Barros *et al*. 2023; Silva *et al*. 2023; Gonçalves *et al*. 2024). The amount of mucilage produced, as well as the nature, size and number of production sites, vary between genera and species of Malvaceae *s.l.* (Metcalfe and Chalk 1950; Krapovickas and Cristóbal 1965; Bouchet 1971; Bouchet and Deysson 1976; Gregory and Baas 1989; Bakker and Gerritsen 1992; Bakker and Bass 1993; Classen *et al*. 1998; Pakravan *et al*. 2007; Rocha *et al*. 2011; Kotina *et al.* 2017; Raghu *et al*. 2019; Silva *et al.* 2023). Secretory idioblasts are solitary cells that differ markedly from neighbouring cells in shape, size and content (Evert 2006). Such cells vary from rounded to axially elongated in shape, are larger, and their secretions can be stored in protoplasmic and apoplastic compartments (Fahn 1979).

Although the cytology of mucilage-secreting idioblasts is relatively well known in Malvaceae species (Bakker *et al*. 1991; Bakker and Gerritsen 1992; Bakker and Bass 1993; Classen *et al*. 1998; Raghu *et al.* 2019), several morphological and developmental aspects remain poorly understood. Studies focussing on this issue can provide important information for ecological, taxonomic and phylogenetic purposes and present additional economic value when considering the substances they produce (Shaheen *et al*. 2009). Here we stress the value of *Peltaea polymorpha*, a Malvaceae subshrub species endemic to Brazil, to study such morphological and developmental aspects of mucilage-secreting idioblasts. This species, which is commonly found in the Cerrado (Brazilian Savanna) of Brazil’s Southern, Southeastern, and Midwestern regions (Fernandes Júnior 2020), has internal secretory structures of mucilage that do not fit typical isolated idioblasts, secretory canals or cavities (personal observations). In this study, we describe a novel component of the mucilage-secretory apparatus based on developmental analysis under light and TEM.

## Material and Methods

### Study site and plant material

This study was performed in a fragment of Cerrado *sensu stricto* located in Botucatu city (22º53ʹ25″S and 48º27ʹ19″W), São Paulo State, Brazil. Cerrado soils are sandy, dystrophic, acidic, have high levels of aluminium, and the vegetation adapts to xeric conditions (Franco 2002). The average annual temperature is 21.34 °C, and the average annual precipitation is 1500 mm in the region. According to the Köppen climate classification, the region’s climate has recently been classified as Aw, with hot, humid summers and cold, dry winters (Franco *et al*. 2023).

Samples of shoot apex, young stem (0.5, 1.0 and 2.0 cm below the apex) and fully expanded leaves located at the third stem node were collected from three adult individuals of *P. polymorpha* growing in the field. Vouchers with reproductive material were deposited in the BOTU Herbarium at the Institute of Biosciences of Botucatu, São Paulo State University (UNESP), Brazil.

### Histology

Plant material was fixed in a solution of 37 % formaldehyde, glacial acetic acid, and 50 % ethanol (Johansen 1940). Some samples were dehydrated in an ethanol series and embedded in a methacrylate resin (Leica, Germany). Cross and longitudinal sections (5 µm) were obtained using a rotating microtome. The sections were stained with 0.05 % toluidine blue pH 4.7 (O´Brien *et al*. 1964) and permanent slides were mounted with synthetic resin Entellan (Merck, Germany). Another portion of the material was manually sectioned using a razor blade. Sections were stained with astra blue and safranin O (Bukatsch 1972) and mounted between slides and coverslips using glycerine. The samples were analysed using a BX 41photomicroscope (Olympus, Japan) and the results were recorded using an attached C7070 digital camera (Olympus, Japan).

### Histochemical tests

Freshly collected samples were manually sectioned using a razor blade and treated with Sudan IV to identify total lipids (Johansen 1940), 10 % ferric chloride solution for phenolic compounds (Johansen 1940), Wagner’s reagent for alkaloids (Furr and Mahlberg 1981), ruthenium red solution for polysaccharides and pectin (Johansen 1940), and 5 % tannic acid and 3 % ferric chloride solution for mucilage (Pizzolato and Lillie 1973). A control test was conducted for each technique according to the descriptions of the techniques proposed by the respective authors.

### Transmission electron microscopy

Samples of young stems were fixed in 2.5 % glutaraldehyde in 0.1 M phosphate buffer (pH 7.3) and post-fixed with 1 % osmium tetroxide in the same buffer for 1 h. Samples were then dehydrated in an acetone series and embedded in Araldite resin (Machado and Rodrigues 2004). The ultrathin sections were contrasted with uranyl acetate and lead citrate (Reynolds 1963). The material was analysed using a Tecnai Spirit transmission electron microscope (FEI Company, USA) at 80 kV.

## Results

**Figure 1.**
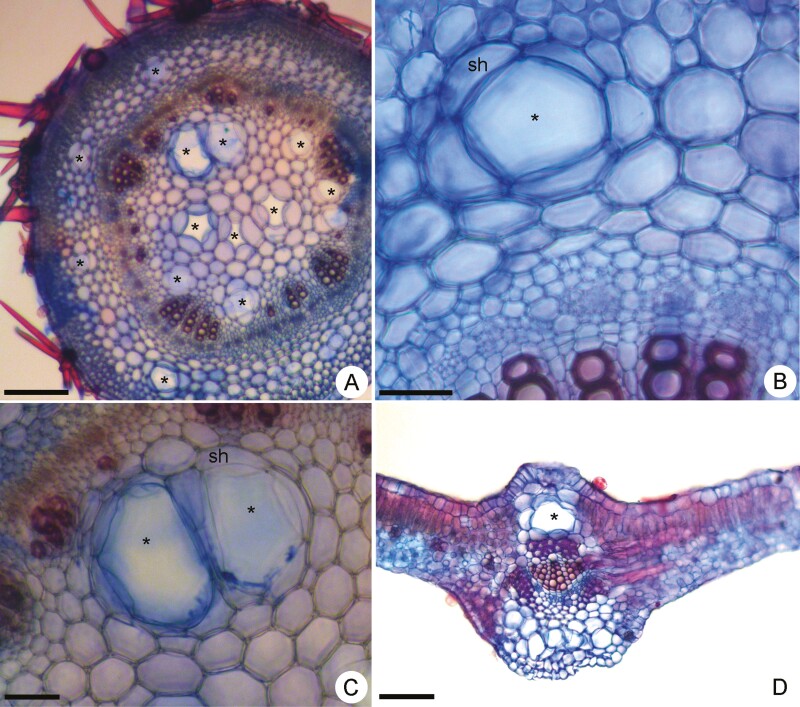
Light micrographs of vegetative organs of *Peltaea polymorpha* in cross sections showing mucilage cells (*) in the cortex (A, B) and pith (C) of stem and in the cortex of leaf midrib (D). sh: sheath parenchyma cells. Scale bars: A, D = 200 µm; B = 50 µm and C = 100 µm.

Secretory cells in *P. polymorpha* were abundant in both the cortex (Fig. 1A and B) and pith (Fig. 1A and C) of the stem and petiole, as well as in the midrib (Fig. 1D) of the leaf blade. Mucilage was histochemically detected in these cells in both leaves and stems. In the cross sections, isolated or clustered mature mucilage cells exhibited a spherical shape (Fig. 1A–D). They were voluminous and surrounded by a sheath of parenchymal cells (Fig. 1B and C). Adjacent mucilage cells occurred very close to each other and remained separated exclusively by a few layers of parenchymal cells (Fig. 1A and C).

**Figure 2.**
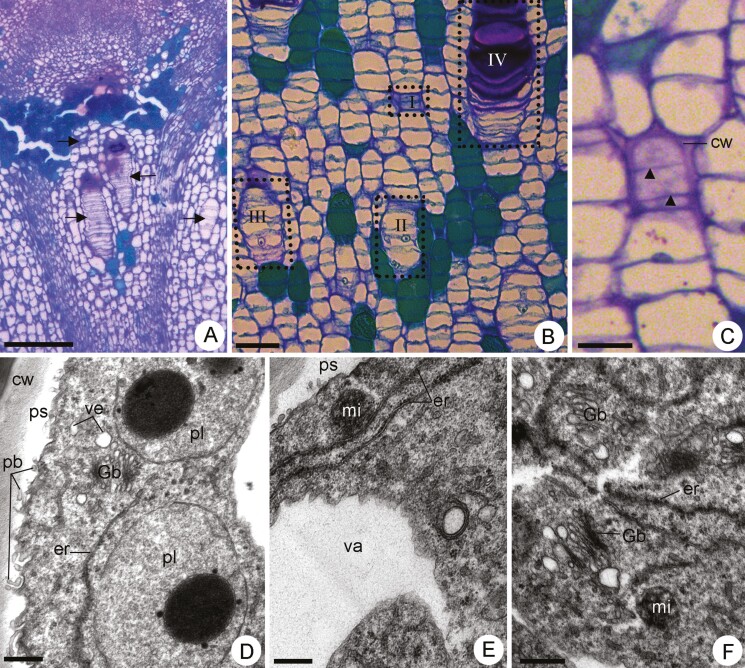
Light micrographs (A–C) and transmission electron micrographs (D–F) of differentiating mucilage cells in *Peltaea polymorpha* shoot apex. (A–C) Longitudinal sections of the shoot apex. (A) Mucilage cells (arrows) in different developmental stages just below the apical meristem. (B) Detail exhibiting rows of mucilage cells in developmental stages I, II, III and IV. (C) Developing mucilage cells in stage I. Observe the thicker lateral walls (cw), thin transversal walls (arrow heads) and dense cytoplasm. (D–F) Differentiating mucilage cells in the developmental stage I. (D) Part of the cell with plasmalemma with irregular contour, abundant paramural bodies (pb) in the periplasmic space (ps) and dense cytoplasm with polyribosomes, Golgi bodies (Gb), vesicles (ve), large plastids (pl) and endoplasmic reticulum (er). (E) Detail showing extensive endoplasmic reticulum (er), vesicles and polyribosomes in the cytoplasm. (F) Abundant and proliferative Gb with attached vesicles. cw: cell wall; er: endoplasmic reticulum; mi: mitochondria; ps: periplasmic space; va: vacuole. Scale bars: A = 200 µm; B = 50 µm; C = 25 µm; D, F = 1 µm and E = 2 µm.

Rows of cells in different developmental stages were observed side-by-side just below the apical meristem (Fig. 2A and B). The internal mucilage-secreting cells in *P. polymorpha* originated early during stem and leaf development from the ground meristem (Fig. 2A and B). A precursor cell (stage I) underwent mitotic divisions originating daughter cells longitudinally arranged (Fig. 2B and C). These precursor cells were isodiametric to slightly tangentially elongated and had thicker lateral walls, thin transverse walls and dense cytoplasm (Fig. 2C). The plasmalemma was irregular in contour, and abundant vesicles were observed near or attached to it (Fig. 2D). Abundant paramural bodies were observed in the developing periplasmic spaces (Fig. 2D and E). The cytoplasm was abundant and the vacuome was reduced (Fig. 2D–F). The large plastids were devoid of thylakoids and contained dark inclusions (Fig. 2D). Abundant and extensive profiles of rough endoplasmic reticulum, proliferative Golgi bodies (GB), globular mitochondria, Golgi derivative vesicles of different sizes and polyribosomes were observed in the cytoplasm (Fig. 2D–F).

**Figure 3.**
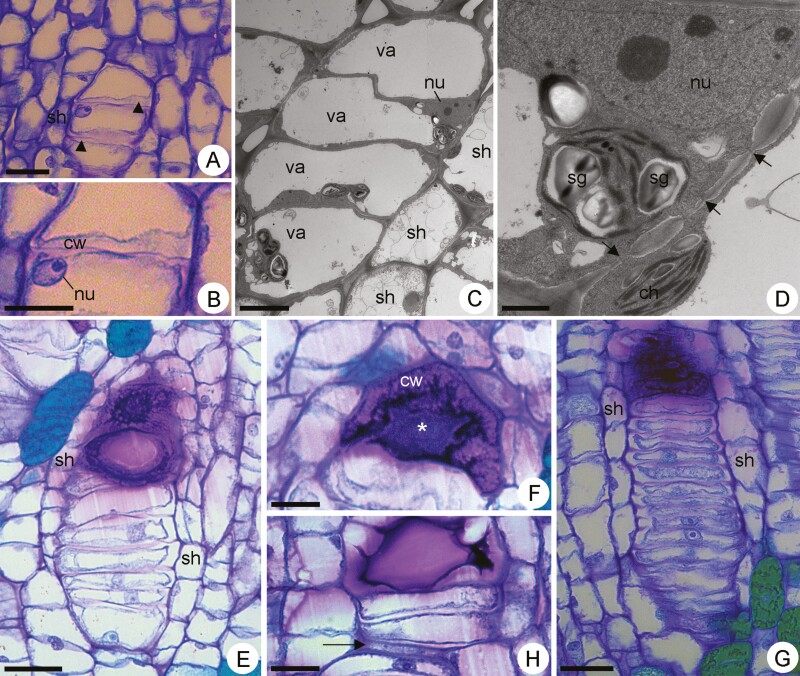
Light micrographs (A–B, E–H) and transmission electron micrographs (C–D) of mucilage cells in different developmental stages in *Peltaea polymorpha* shoot apex. (A) Longitudinal section showing differentiating mucilage cells in stage II with swollen transversal walls. (B) Detail of the previous figure showing swollen transversal walls (cw) and nucleus (nu). (C) Rowed differentiating mucilage cells in stage II exhibiting developed vacuoles (va) and reduced cytoplasm. (D) Detail of the previous figure showing differentiating mucilage cells with a large nucleus (nu) and plastids containing starch grains (sg). Primary pit fields with plasmodesmata (arrows) connect the mucilage cells to the neighbouring sheath parenchyma cells. Observe typical chloroplast (ch) in the sheath parenchyma cell. (E–F) Longitudinal view showing developing mucilage cells in stage III. (E) Observe accumulation of mucilage in the more distal cells of the row. (F) Mucilage cells with mucilage accumulated in cell walls (cw) and protoplast (*). (G–H) Developing mucilage cells in stage IV. (G) Row of narrow differentiating mucilage cells surrounded by parenchyma sheath (sh). (H) Detail showing flattened cell (arrow) in a row due to crushing caused by the enlargement of the neighbouring cells. Scale bars: A, E, G = 50 µm; B, F, H = 20 µm; C = 15 µm and D = 5 µm.

In stage II, the differentiating mucilage cells were arranged in rows of up to four cells (Fig. 3A–C). These cells began to increase in volume and their transverse walls became swollen (Fig. 3A and B). Accumulation of polysaccharide material was observed in the cell walls (Fig. 3A and B). The vacuoles increased in volume and the cytoplasm was reduced to a very straight peripheral layer (Fig. 3C). Their nuclei were large, with small lumps of heterochromatin and the nucleolus was evident (Fig. 3A–D). Plastids remained voluminous and exhibited thylakoids and large starch grains (Fig. 3D). The neighbouring common parenchyma cells became juxtaposed at some points around the row of differentiating mucilage cells, beginning an arrangement to form a sheath-like envelope (Fig. 3A and C). These parenchyma sheath cells were irregular in shape and size and exhibited vacuoles (Fig. 3C) and typical ellipsoid chloroplasts (Fig. 3D). Primary pit fields with evident plasmodesmata connected the rowed differentiating idioblasts between themselves and sheath parenchyma cells (Fig. 3D).

Mitotic divisions continued to occur, increasing the number of developing cells in a row. In stage III, the rows had about 10 cells that became progressively tangentially elongated and narrow, acquiring a rectangular shape, excluding the most distal and the most basal cells of the rows that remained slightly isodiametric (Fig. 3E). In this stage, some cells of the row, especially the most distal ones, started to exhibit denser content that was histochemically identified as mucilage (Fig. 3E). The transversal walls of such cells swelled and compressed the cytoplasm (Fig. 3E). Mucilage was accumulated in both cell walls and protoplast (Fig. 3F). In this stage, the parenchyma sheath was better defined and almost completely involved the row of developing mucilage cells (Fig. 3E).

In stage IV, the rows were constituted by about 20 differentiating mucilage cells (Fig. 3F) that became even narrower (Fig. 3G and H). Some of them became very flattened due to crushing caused by the enlargement of the neighbouring cells (Fig. 3H). At this stage, the row of differentiating mucilage cells was totally surrounded by a sheath of parenchyma cells (Fig. 3G) devoid of secretory activity.

**Figure 4.**
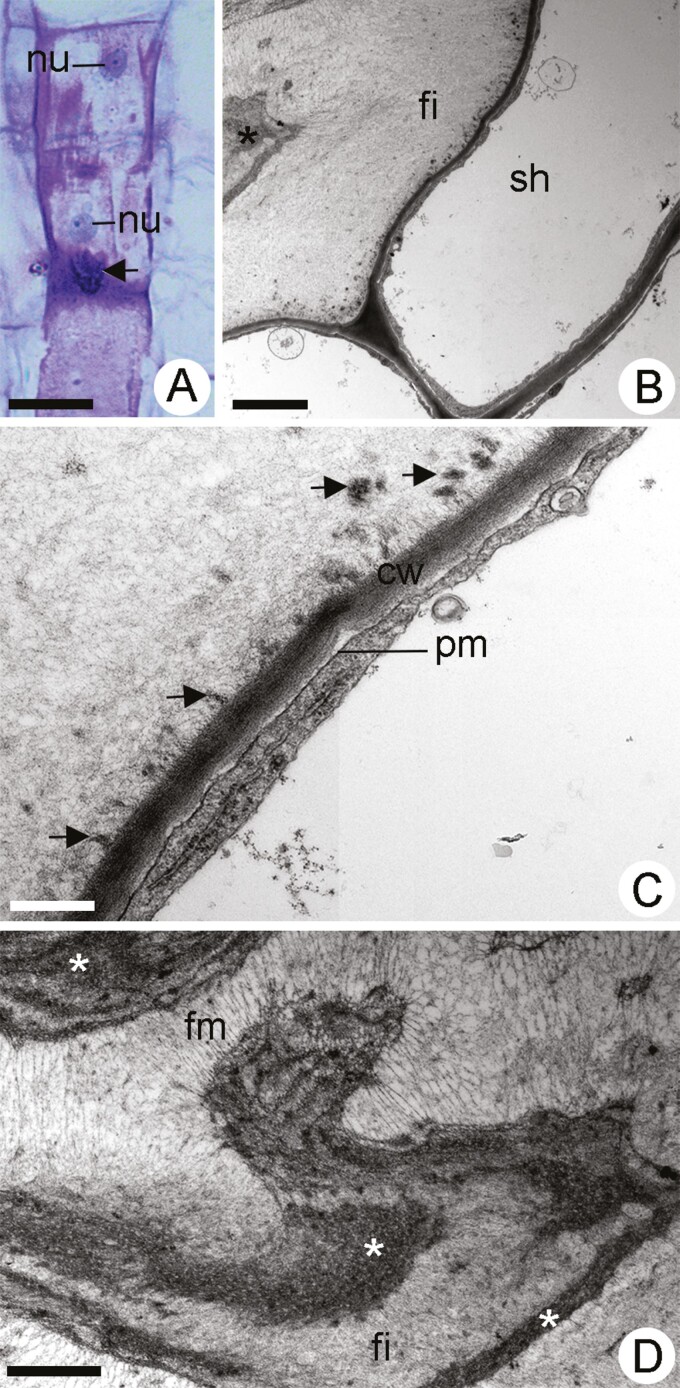
Light micrography (A) and transmission electron micrographs (B–D) of differentiating mucilage cells in stage V in *Peltaea polymorpha* stem. (A) Longitudinal view of rowed developing mucilage cells exhibiting scattered accumulation of mucilage. Observe mucilage lumps (arrow) in the vicinity of the cell walls. (B) Fibrillar material (fi) in mucilage cell pushing the cytoplasm (*) toward the inner portion of the cell. (C) Portion of mucilage cell showing lumps of dense material (arrows) detached from the walls (cw) and mixed with mucilaginous secretion. Observe plasmalemma (pm) in the sheath parenchyma cell. (D) Mucilage cell with cytoplasmic strands (*) immersed in a mucilaginous matrix with a fibrillar (fi) appearance. Observe the net of filamentous material (fm) connecting remnants of cytoplasm. Scale bars: A = 25 µm; B = 10 µm; C = 1 µm and D = 2 µm.

In the elongating internodes of stem, the rowed differentiating mucilage cells in stage V underwent stretching, exhibiting a square to slightly elongated longitudinally shape (Fig. 4A). Voluminous, spherical nuclei with nucleoli remained presented in each rowed cell (Fig. 4A). Mucilage appeared to accumulate in a generalized manner and was observed throughout the cellular volume as a mass of pink-stained material using toluidine blue under light microscopy (Fig. 4A). Mucilage lumps were seen in the vicinity of the cell walls (Fig. 4A). Ultrastructurally, a fibrillar material was scattered through the mucilage cells pushing the cytoplasm toward the inner region of the cell (Fig. 4B). Lumps of dense fibrillar material detached from the lateral walls and mixed with mucilaginous secretions (Fig. 4C). The plasmalemma was not visualized (Fig. 4C), and the organelles were confined in cytoplasmic strands immersed in a mucilaginous matrix with a fibrillar appearance (Fig. 4D). A net of fine filamentous material, resembling actin microfilaments, was commonly observed connecting remnants of cytoplasm to the cell wall (Fig. 4D). The neighbouring sheath parenchyma cells exhibited an intact plasmalemma, a well-developed vacuome and reduced peripheral cytoplasm, with no evidence of secretory activity (Fig. 4B and C).

**Figure 5.**
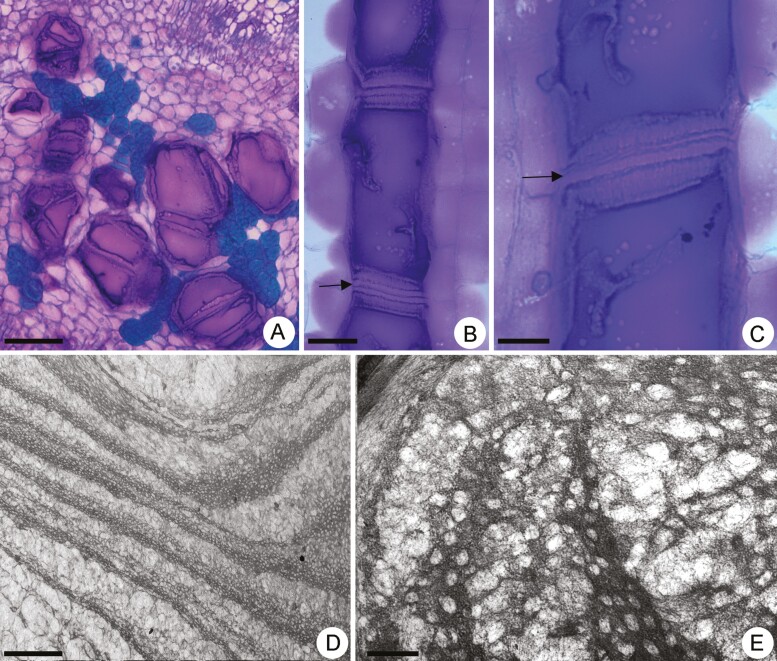
Light micrographs (A–C) and transmission electron micrographs (D–E) of differentiating mucilage cells in stage VI in *Peltaea polymorpha* stem. (A). Cross section showing mucilage cells filled with homogenous material. (B–C) Longitudinal view showing mucilage cells with swollen and multilayered transverse walls. The arrows indicate reminiscent of flattened cells. (D) Portion of mucilage cells showing transversal wall with multilayer appearance with alternating electron-dense layers and lighter layers. (E) Detail of the transversal wall showing denser layers with smaller pores and cellulose microfibrils with a more compact arrangement and clearer layers with enlarged pores and cellulose microfibrils loosely arranged. Scale bars: A = 100 µm; B = 50 µm; C = 20 µm; D = 5 µm and E = 1 µm.

In the mature internodes of the stem, the mucilage cells in stage VI were filled with homogenous content that was dark stained with toluidine blue (Fig. 5A–C). Their transverse walls were highly swollen and multilayered (Fig. 5B and C). The walls whose cells had undergone flattening at earlier stages of development remained in the row (Fig. 5B and C). This phenomenon resulted in a sandwich panel structure consisting of the swollen transversal walls of adjacent cells visualized in some portions of the row of mucilage cells (Fig. 5B and C). Ultrastructurally, these walls exhibited numerous pores arranged in alternating layers of different densities (Fig. 5D). In the denser layers, the pores were smaller and immersed in a matrix of cellulose microfibrils with a fibrous appearance (Fig. 5D and E). In the clearer layers, the pores were enlarged and the cellulose microfibrils were loosely arranged (Fig. 5D and E).

**Figure 6.**
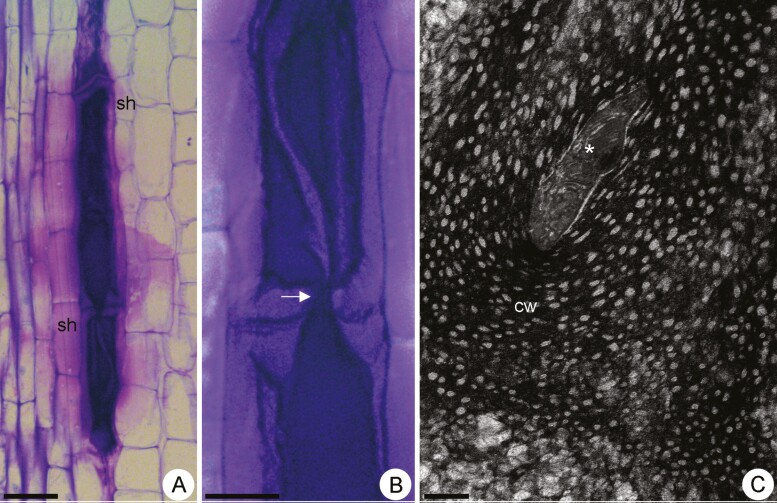
Light micrographs (A, B) and transmission electron micrograph (C) of mucilage cells in stage VII in *Peltaea polymorpha* stem. (A) Longitudinal view showing rowed mucilage cells containing mucilage in both cell wall and protoplast. (B) Detail of the previous figure showing a strand of cytoplasm (white arrow) crossing the transversal walls and connecting two-rowed mucilage cells. (C) Strand of cytoplasm (*) with reminiscent of organelles amidst the porous walls (cw). sh: parenchyma sheath. Scale bars: A = 100 µm; B = 20 µm and D = 2 µm.

In stage VII, the cells were more stretched longitudinally (Fig. 6A) and mucilage was accumulated in a generalized manner in both cell wall and protoplast (Fig. 6A and B). The terminal cells of a row exhibited tapered ends, resembling intrusive growth (Fig. 6A). Strands of cytoplasm crossed the transverse walls and connected the rowed mucilage cells (Fig. 6A and B). Reminiscent organelles were observed in these cytoplasmic strands amid the porous walls (Fig. 6C). The sheath of juxtaposed common parenchyma cells remained enveloping those rowed interconnected mucilage cells (Fig. 6A and B).

## Discussion

In this study, we showed that the internal mucilage-secretory system in *Peltaea polymorpha* is characterized by a row of initially flattened cells that extend axially during their development following the stem elongation. These cells become filled with mucilage and communicate between themselves by cytoplasmic strands in mature stages, characterizing interconnected idioblasts.

Conventionally, idioblasts are solitary cells differing in form, size and contents from the neighbouring cells in the same tissue (Foster 1956). Otherwise, in *P. polymorpha,* mature mucilage idioblasts form a row of cells interconnected by cytoplasmic strands. This connection is achieved by the subcellular organization of the transversal cell walls characterized by a fibrillar structure with multiple pores that allowed streaming of the cytoplasm. Cytoplasmic strands are thought to function as transport routes for substances and organelles and involve the action of the cytoskeleton, especially the actin filament (van der Honing *et al*. 2007). In fact, a net of fine fibrillar material, resembling actin microfilaments, was here observed connecting remnants of cytoplasm to the cell wall in *P. polymorpha* (see Fig. 4D). Therefore, the remarkable occurrence of cytoplasmic strands between rowed idioblasts is evidence of the symplastic continuity between the mucilage cells, forming, in the end, a long and continuous secretory system. This phenomenon is distinct of the described in other plant species where contiguous mucilage cells form a secretory system resulting from the dissolution of the adjoining walls (Classen *et al*. 1998; Barros *et al*. 2023). Cell wall dissolution between contiguous mucilage cells was not observed in *P. polymorpha*. Thus, the ontogenesis of the internal secretory system in *P. polymorpha* does not involve fusion by cell lysis or cell wall breakage, in contrast to the previous descriptions of Malvaceae (Bakker and Gerritsen 1992; Bakker and Baas 1993; Classen *et al*. 1998; Barros *et al.* 2023; Gonçalves *et al*. 2024).

Despite their elongated appearance in the longitudinal plane, secretory structures in *P. polymorpha* are formed by rowed interconnected idioblasts, which excludes the possibility of their classification as secretory canals. Our developmental analysis showed that neighbouring common parenchymal cells rearranged around secretory idioblasts during their ontogenesis. It is important to note that the presence of a sheath of parenchyma cells around the mature rowed mucilage cells in *P. polymorpha* resembles secretory canals at first sight. However, idioblasts and sheath parenchyma cells are originated from distinct precursor cells. In addition, the sheath parenchyma cells do not have ultrastructural characteristics associated with the synthesis, accumulation, or release of secretions, preventing their characterization as epithelial cells. In this sense, other studies highlighted the difficulty of correctly classifying mucilage-secretory structures due to the physicochemical properties of the secretion itself, stressing the need for detailed ontogenetic studies (Barros *et al*. 2023; Gonçalves *et al*. 2024). Ultrastructurally, the idioblasts in *P. polymorpha* exhibited typical features of mucilage-secretory cells, such as large nuclei with evident nucleolus, large amount of endoplasmic reticulum, abundant GB and vesicles and plastids devoid of thylakoids, as reported for different mucilage-secretory systems (Trachtenberg and Fahn 1981; Evert 2006; Barros *et al*. 2023) and consistent with mucilage production (Evert 2006).

The widespread accumulation of mucilage in both cell walls and protoplast leads to an increase in the volume of the secretory cells active in secretion, which can lead to the crushing of some cells in the row, resulting in a sandwich panel structure consisting of the swollen transversal walls of adjacent cells. The swelling of the mucilage cell walls, especially the transverse walls, is noticeable in *P. polymorpha*. In this species, we observed the formation of a multilamellar and porous structure in the transverse walls of the mucilage idioblasts. This wall structure seems to reflect the formation of a network of microchannels, which has been attributed to the presence of hygroscopic compounds that could maintain leaf hydration and promote water uptake (Losada *et al*. 2021). Based on our observations, we suggest that the swelling of the walls leads to the compression of the cytoplasm, which would cause the formation of strands of cytoplasm through the wall, as previously discussed, which is facilitated by its porous structure.

From a developmental point of view, this study has provided information on the biology of mucilage-secreting cells and has shown a new type of mucilage idioblast structure, which we call interconnected idioblasts. Developmental analyses were crucial for understanding the morphological changes that culminate in the fully differentiated idioblasts. Further ultrastructural studies on these cells could help us learn more about mucilage-secreting cells in general and determine their functional properties. Besides, we can achieve further improvements in our understanding of the biology and function of mucilage-secreting cells by (i) broadening the number of Malvaceae species from a phylogenetic perspective and (ii) using convergent study techniques (e.g. live fluorescence imaging, advanced electron microscopy, such as tomography, and immunolocalization studies) to elucidate many aspects of subcellular organization that remain incompletely understood. Our findings open new avenues for understanding the structure and dynamics of mucilage-secreting cells from a functional perspective while elucidating the intriguing morphological nature of the internal mucilage-secretory system in *P. polymorpha*.

## Data Availability

The data analysed are included in this manuscript.
